# How Very-Long-Chain Fatty Acids Could Signal Stressful Conditions in Plants?

**DOI:** 10.3389/fpls.2016.01490

**Published:** 2016-10-18

**Authors:** Antoine De Bigault Du Granrut, Jean-Luc Cacas

**Affiliations:** ^1^UMR1318 Institut National de la Recherche Agronomique-AgroParisTech, Centre Institut National de la Recherche Agronomique de Versailles-Grignon, Institut Jean-Pierre BourginVersailles, France; ^2^Département Sciences de la Vie et Santé, AgroParisTech, UFR de Physiologie VégétaleParis, France

**Keywords:** very-long-chain fatty acids, biotic and abiotic stress, signaling cascades, sphingolipids, membrane microdomains, plasma membrane, endoplasmic reticulum, secretory pathway

## Abstract

Although encountered in minor amounts in plant cells, very-long-chain fatty acids exert crucial functions in developmental processes. When their levels are perturbed by means of genetic approaches, marked phenotypic consequences that range from severe growth retardation to embryo lethality was indeed reported. More recently, a growing body of findings has also accumulated that points to a potential role for these lipids as signals in governing both biotic and abiotic stress outcomes. In the present work, we discuss the latter theory and explore the ins and outs of very-long-chain fatty acid-based signaling in response to stress, with an attempt to reconcile two supposedly antagonistic parameters: the insoluble nature of fatty acids and their signaling function. To explain this apparent dilemma, we provide new interpretations of pre-existing data based on the fact that sphingolipids are the main reservoir of very-long-chain fatty acids in leaves. Thus, three non-exclusive, molecular scenarii that involve these lipids as membrane-embedded and free entities are proposed.

## Introduction

Both abiotic and biotic stresses, as well as developmental cues, have long been known to drastically modify lipid composition—including fatty acid (FA) content—at the organ level. For instance, it is well-documented that phosphate starvation reorients lipid anabolism from phospholipid toward galactolipid synthesis (Kobayashi et al., 2006), likely for maintaining plant cell homeostasis until the constraint is relieved. Likewise, temperature-induced stress provokes changes in plasma membrane (PM) physico-chemical properties due to modification of sterol concentration and FA double bond index (Los and Murata, 2004). Progressive loss of chloroplast galactolipids is also a well-defined hallmark of foliar senescence processes (Jia and Li, 2015). Furthermore, plant resistance to pathogens can cause the consumption of chloroplast-originating polyunsaturated fatty acids for supplying an oxidative pathway that orchestrates host cell dismantling (Cacas et al., 2005). Obviously, all these events, whether or not associated with stress acclimation, are relevant to profound structural alterations and mobilize huge amounts of lipids that can be readily quantified by regular biochemical methods. By contrast, one can easily imagine that lipid-contingent signaling events relies on more subtle changes. This is perfectly illustrated by the case of phosphatidic acid, a conserved stress signaling molecule produced by either phospholipase D or the coordinated action of phospholipase C and diacylglycerol kinase (Guo et al., 2011). Because of its low abundance, phosphatidic acid is commonly evidenced by *in vivo* isotopic labeling experiments (Arisz et al., 2009; Cacas et al., 2016a). Another example that could be cited is that of the FA-derived hormonal signal jasmonic acid that requires highly sensitive liquid chromatography-based methods for efficient quantification (Glauser and Wolfender, 2013; Cacas et al., 2016b). Additionally, to the best of our knowledge, marked degradation of the respective lipid substrates alimenting the two latter signaling cascades were rarely correlated with signal generation. Hence, this hints the importance of carefully considering, whenever possible, absolute concentrations of metabolites involved when discriminating among signaling events and structural changes. What about very-long-chain fatty acids (VLCFA)? How are they synthesized? And, how their levels are affected in response to stress?

## Biosynthesis of very-long-chain fatty acids in plant cells

In plants, lipid metabolism is highly compartmentalized and this intricate organellar networks allows fine-tuned regulation of the intracellular catabolic/anabolic balance for approximately several thousands of molecular lipid species. Biosynthesis of FA-containing lipids—mostly phospholipids, galactolipids, sphingolipids, triacylglycerides, and to a lesser extent, acylsteryl-glycosides—relies on two interacting metabolic routes: the “*prokaryotic pathway”* that resides in plastids and the “*eukaryotic pathway*” that is localized to endoplasmic reticulum (ER). Basically, production of FA-building units is initiated in plastids by the fatty acid synthase (FAS) complex II that uses malonyl-CoA and acetyl-CoA as co-substrates and NADPH as reductant. Each FAS-mediated cycle adds 2 carbons to acyl-CoA chains until molecules reaches a length of 16 or 18 carbons. Combined thioesterase and acyl-CoA synthetase activities are then invoked in active export of aliphatic chains from stroma to cytoplasm, where this pool of activated molecules is used by the ER for further chain length extension (Li-Beisson et al., 2010).

**Figure 1:**
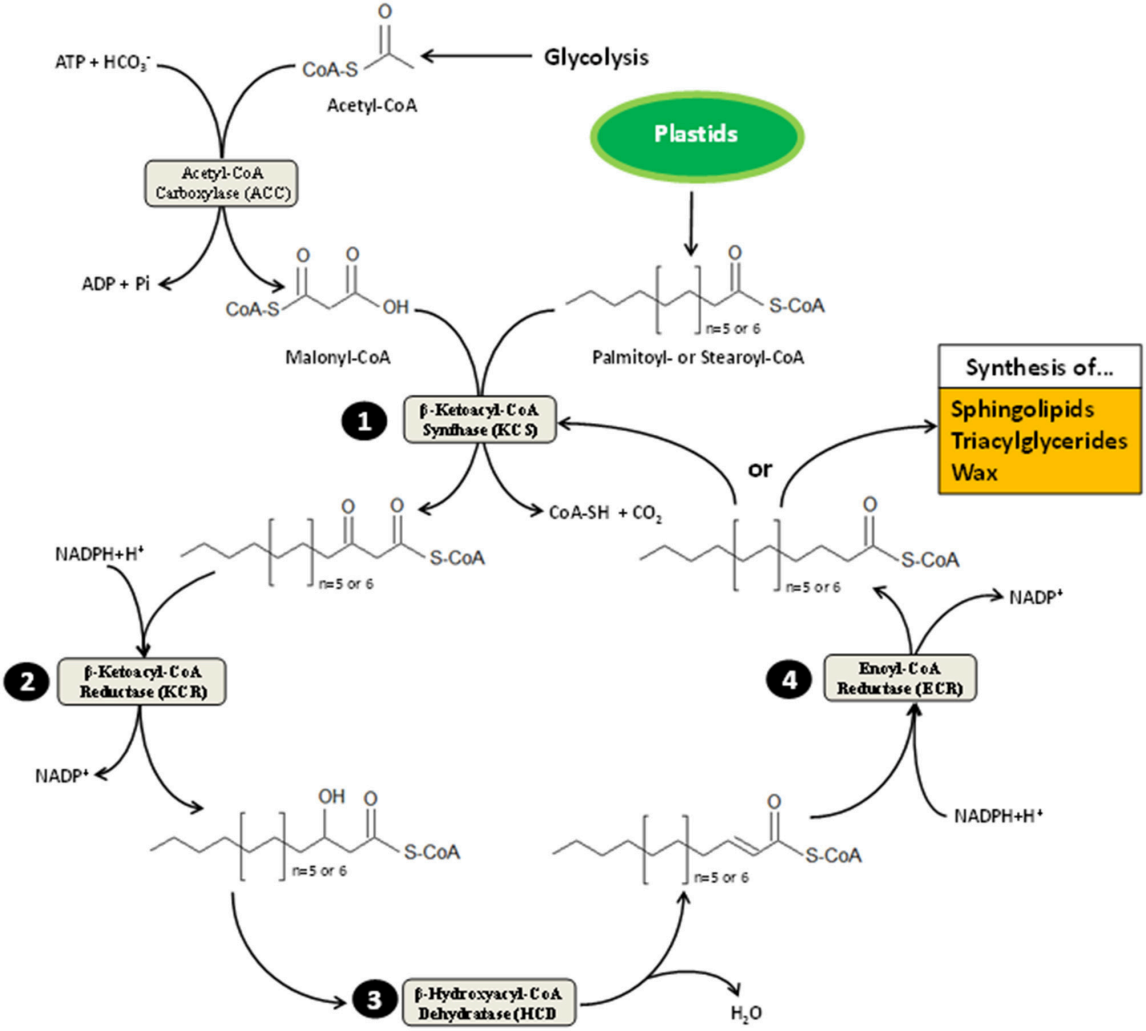
**Scheme representation of very-long-chain fatty acid elongation**. This process takes place on cytosolic side of the ER membrane. It is alimented by acetyl-CoA and acyl-CoA originating from the cytoplasm-located glycolysis and plastid-resident FA elongation pathway, respectively. Plastids provides 16 or 18 carbon-long acyl-CoA (i.e., palmitoyl- and stearoyl-CoA) to be elongated. The first committed step to VLCFA elongation cycle is catalyzed by the β-ketoacyl-CoA synthase (KCS), which condenses malonyl-CoA (synthesized by carboxylation of acetyl-CoA moieties) with palmitoyl- or stearoyl-CoA. Resulting products are then reduced into β-hydroxacyl-CoA (step 2) before losing a molecule of water (step 3); the latter reaction of which is mediated by the β-hydroxacyl-CoA dehydratase (HCD). Upon dehydration, β-enoyl-CoA undergo reduction (step 4), forming acyl-CoA that harbor two additional carbons. These products can either be oriented toward sphingolipid, wax and triacylglyceride synthesis depending on tissue specificity and cell requirement or reenter VLCFA elongation cycle until its length reaches 28 carbons in *Arabidopsis* or more in other plant species. Each cycle turn consumes one molecule of ATP and two of NADPH+H^+^.

Very-long-chain fatty acids, formally defined as FA longer than 18 carbons, are extended by an ER membrane-embedded protein complex of 4 enzymes, acting presumably on the cytosolic side (see Haslam and Kunst, 2013 for an updated review). Fatty acid elongase activity results in successive action of β-ketoacyl-CoA synthase (KCS), β-ketoacyl-CoA reductase (KCR), β-hydroxyacyl-CoA dehydratase (HCD), and enoyl-CoA reductase (ECR). Each of these enzymes utilizes as substrate the product of the previous one in cycles beginning by malonyl-CoA condensation to long-chain acyl-CoA (Figure 1). Except for ECR, which is a single copy-encoded gene in *Arabidopsis thaliana*, a huge multigenic family composed of 21 members codes for tissue-specific KCS enzymes (Joubès et al., 2008) that are thought to dictate the length of acyl-CoA chains produced by the complex (Fehling and Mukherjee, 1991; Millar and Kunst, 1997). Both KCR and HCD are encoded by 2 independent genes, dubbed *KCS1/KCS2* and *PASTICCINO2/PTPLA*, respectively (Table 1). Such a complexity could suggest that multiple elongase complexes, which differ by their relative composition, coexist in ER membranes. In other words, functionally spatialized-domains with large metabolon units could orient the lipid class into which very-long-acyl chains are incorporated. But, only indirect evidence for this kind of ER sub-compartmentation were reported so far (Shockey et al., 2006). VLCFA are mainly present in the impermeable cuticular wax layer deposited at the plant aerial organ surface, in triacylglycerides found in seed oil and in sphingolipids, which act as structural elements in lipid bilayers forming endomembranes and PM (Bach and Faure, 2010).

**Table 1:** **Nomenclature of the VLCFA elongase complex-encoding genes**.

**Gene names**	**Other gene names**	**Loci (AGI)**	**Protein activity**	**Protein length (aa)**	**M.W. (kDa)**	**pI**
*KCS1*	-	At1g01120	β-ketoacyl-CoA synthase (catalyzes the first committed step to VLCFA synthesis)	528	59.28	8.9
*KCS2*	-	At1g04220		528	59.53	9.6
*KCS3*	-	At1g07720		478	54.33	9.5
*KCS4*	-	At1g19440		516	57.84	9.1
*KCS5*	*CER60*	At1g25450		492	55.65	8.9
*KCS6*	*CER6, CUT1, POP1*	At1g68530		497	56.40	9.1
*KCS7*	-	At1g71160		460	51.50	8.3
*KCS8*	-	At2g15090		481	54.19	9.4
*KCS9*	-	At2g16280		512	57.97	9.4
*KCS10*	*FDH*	At2g26250		550	61.96	9.3
*KCS11*	-	At2g26640		509	57.81	9.6
*KCS12*	-	At2g28630		476	53.97	9.0
*KCS13*	HIC	At2g46720		466	52.18	9.3
*KCS14*	-	At3g10280		459	51.63	9.4
*KCS15*	-	At3g52160		451	51.11	9.7
*KCS16*	-	At4g34250		493	55.78	9.1
*KCS17*	-	At4g34510		487	54.91	9.7
*KCS18*	*FAE1*	At4g34520		506	56.26	9.8
*KCS19*	-	At5g04530		464	52.61	8.6
*KCS20*	-	At5g43760		529	59.31	9.2
*KCS21*	-	At5g49070		464	52.56	9.3
*KCR1*	-	At1g67730	ketoacyl-CoA reductase	318	35.76	9.9
*KCR2*	-	At1g24470		312	35.00	9.8
*HCD*	*PAS2*	At5g10480	β-hydroxyacyl-CoA dehydratase	230	26.41	9.7
*PTPLA*	-	At5g59770		272	30.96	10
*ECR*	*CER10, GLH6*	At3g55360	enoyl-CoA reductase	310	35.72	9.7

*This table provides information on regular gene names, additional designations found in the literature and referenced loci based on the Arabidopsis Genome Initiative (AGI). Demonstrated or potential enzymatic activities of the corresponding proteins are also indicated. Protein length is expressed as the number of amino acids (aa). M.W. and pI refers to molecular weight and isoelectric point, respectively. Most data were retrieved from The Arabidopsis Information Resource (TAIR) website (https://www.arabidopsis.org/) and crossed with the Arabidopsis book chapter dedicated to acyl-lipid metabolism (Li-Beisson et al., 2010). Abbreviations: CER6, 10 and 60, ECERIFERUM 6, 10 and 60; CUT1, CUTICULAR 1; FAE1, FATTY ACID ELONGATION 1; FDH, FIDDLEHEAD; GLH6, GLASSY HAIR 6; HIC, HIGH CARBON DIOXIDE; PAS2, PASTICCINO 2; POP1, POLLEN-PISTIL INCOMPATIBILITY 1; PTPLA, PROTEIN-TYROSINE PHOSPHATASE-LIKE*.

## Changes in very-long-chain fatty acid levels in plant cells undergoing stress

With respect to modifications of VLCFA concentrations under stressful conditions, sparse data have been obtained but clear trends are currently emerging (Table 2). Overall, numerous abiotic constraints (salt, cold, hypoxia, heavy metal exposure…) were reported to increase VLCFA contents in distinct plant species. Induced *Arabidopsis* resistance to bacterial pathogens seems also associated with an augmentation of endogenous VLCFA levels (Raffaele et al., 2008). Not surprisingly, detailed lipid analysis revealed that VLCFA, which are both components and precursors of epicuticular wax, are affected by drought stress and bacterial infection in proportions which are clearly relevant to structural changes (Raffaele et al., 2008; Zhu and Xiong, 2013). This experimental fact makes full sense as cuticle is involved in limiting stomata-independent evaporation in shoots, suggesting a reinforcement of this hydrophobic layer under water stress. In the context of pathogen invasion, strengthening the apoplastic barrier is also a well-known defense phenomenon (Garcion et al., 2014), believed to prevent further micro-organism penetration and spreading.

**Table 2:** **Changes in VLCFA levels under stressful conditions**.

**Plant models**	**Stresses**	**FA phenotypes**	Affected lipid classes^*^	**Analyzed organs**	**References**
*Nigella sativa* L.	Mild Zn^2+^ exposure	Increase in 20:0, 22:0 and 24:0	n.d.	Seeds	Marichali et al., 2016
		Increase in 20:0, 22:0 and 24:0 Decrease in 20:1	n.d.	Leaves	
		Increase in 20:1, 22:0 and 24:0 Decrease in 20:0	n.d.	Stems	
		Increase in 20:0, 22:0 and 24:0 Decrease in 20:1	n.d.	Roots	
*Noccaea caerulescens*, ecotype Mezica	Cd^2+^ exposure	Decrease 26:0, 28:0 and 30:0 Increase in 20:2 and 20:3	n.d.	-	Zemanová et al., 2015
*Tetraselmis sp*. M8	Salt	Increase in 20:4 and 20:5	n.d.	-	Adarme-Vega et al., 2014
*Artemisia annua*	Long-term salinity	Decrease in 22:0 and 24:0 Increase in 22:1	n.d.	Leaves	Qureshi et al., 2013
*Taxus chinensis* cv. *mairei*	Shear stress	Increase in 20:0, 20:1, 22:0, 24:0 and 25:0	n.d.	Suspension cell culture from stem	Han et al., 2009
Rice (*Oriza sativa*)	Drought	Increase in 26:0 and 28:0	Cuticular wax	Leaves	Zhu and Xiong, 2013
*Arabidopsis thaliana*	Hypoxia	Increase in 22:0, 24:0 and 24:1	GIPC and GluCer	Leaves	Xie et al., 2015
*Arabidopsis thaliana*	Oxidative stress	Increase in total hVLCFA	n.d.	Leaves	Nagano et al., 2012
*Arabidopsis thaliana*	Cold	n.d.	Increase in GIPC	Shoots	Nagano et al., 2014
*Arabidopsis thaliana*	Pst DC3000::AvrRpm1	Total VLCFA	n.d.	Leaves	Raffaele et al., 2008

*This table provides an overview of selected publications dealing with abiotic stress. Of note, to the best of our knowledge, the only published work reporting on biotic stress and VLCFA content is that of Raffaele et al. (2008). Nomenclature for fatty acids (FA) is as follows: the first number indicates the carbon chain length and the second the number of unsaturation (example: 24:1 is 24 carbon-long FA which possesses one double bond). n.d., not determined*,

* *refers to the lipid classes whose VLCFA content is altered. Pst refers to Pseudomonas syringae pv. tomato*.

Pioneering works pointed out the transcriptional activation of genes coding for members of the *Arabidopsis* ER-localized elongase complex in response to stress. It has been demonstrated that multiple KCS-encoding genes were responsive to light conditions, dehydration, salt, cold, and osmotic stresses (Joubès et al., 2008). Mutants deficient for the transcription factor MYB30 were proven to be unable to accumulate WT levels of VLCFA under hypoxia (Xie et al., 2015). In addition, microarray experiments showed that 3 out of the 21 *KCS* genes (*KCS1, KCS2*, and *KCS10*), one *HCD* gene (*PASTICCINO 2*) and the only *ECR* gene (CER10) were transcriptionally up-regulated during incompatible interaction with bacteria, and the consecutive increase in VLCFA levels was confirmed by biochemical approach. This transcriptional reprogramming was further shown to be under the control of MYB30 (Raffaele et al., 2008). Although elongase regulation could account for cuticle structure readjustment, one cannot rule out the possibility that it could reflect an unusual context where VLCFA-contingent changes hide signaling cascades. Arguing in favor of this idea are several lines of evidence. Firstly, concentrations of VLCFA mobilized in many instances described in the literature are all the more sufficient for signaling purposes (Table 2). Secondly, except for drought stress (Zhu and Xiong, 2013), no data can currently explain clearly the role of VLCFA in certain specific abiotic contexts (like cold stress, mechanical injury and others) by the solely bias of cuticle. Thirdly, other lipids than wax components, such as complex sphingolipids that are potential reservoirs of signal molecules (Gronnier et al., 2016), exhibit significant changes in their VLCFA contents following stress (Table 2). Lastly, transgenic lines that displayed VLCFA over-accumulation correlated with enhanced pathogen-contingent cell death phenotype (Raffaele et al., 2008). Given that cuticle-related processes are unlikely to control programmed cell death, it must be envisaged that VLCFA exert their putative effects on cell fate in an alternative manner. Thus, it seems reasonable to investigate the concept that VLCFA could participate to stress signaling pathway.

## Are free very-long-chain fatty acids genuine signaling molecules?

In humans, lipid homeostasis is tightly controlled, and its long-term perturbation can have severe deleterious effects on health. Free FA contribute to the regulation of organ and tissue homeostasis by acting as signaling molecules through autocrine or paracrine cell non-autonomous modes. Extracellular free FA concentrations can be finely perceived by plasma membrane-localized protein receptors that discriminate among chain lengths. These are named FREE FATTY ACID RECEPTORS (FFAR)/G PROTEIN-COUPLED RECEPTORS (GPAR) (Ichimura et al., 2014). In plant genomes, no genes coding for such orthologous receptors could be retrieved by sequence comparison (unpublished data). Besides, even when a number of long-chain acyl-CoA binding proteins (ACBP) were reported to participate in plant stress tolerance (Xiao and Chye, 2011), it seems that they rather function as general regulators of lipid metabolism than as cognate signaling partners of acyl chains in challenged cells. For instance, AtACBP2 and AtACBP4 were found to physically interact with an ethylene-responsive transcription factor (Li and Chye, 2004; Li et al., 2008), possibly controlling by this means lipid-related gene expression. Another issue for VLCFA to be considered as genuine signals relies on their amphipathic nature that renders them strong membrane destabilizers and not prone to cross lipid bilayers. This certainly prevents unmodified VLCFA from functioning as soluble signals at both the intra—and extra-cellular levels. Therefore, alternative hypotheses must be imagined for explaining how these lipids could regulate plant stress responses. What could be the molecular mechanisms invoked? Based on the literature, three potentially interconnected scenarii taking into account the observed relatively high amounts of VLCFA mobilized during stress response are described hereafter.

## “The indirect sphingolipid signaling hypotheses”–how to reconcile very-long-chain fatty acids with stress signaling?

**Figure 2:**
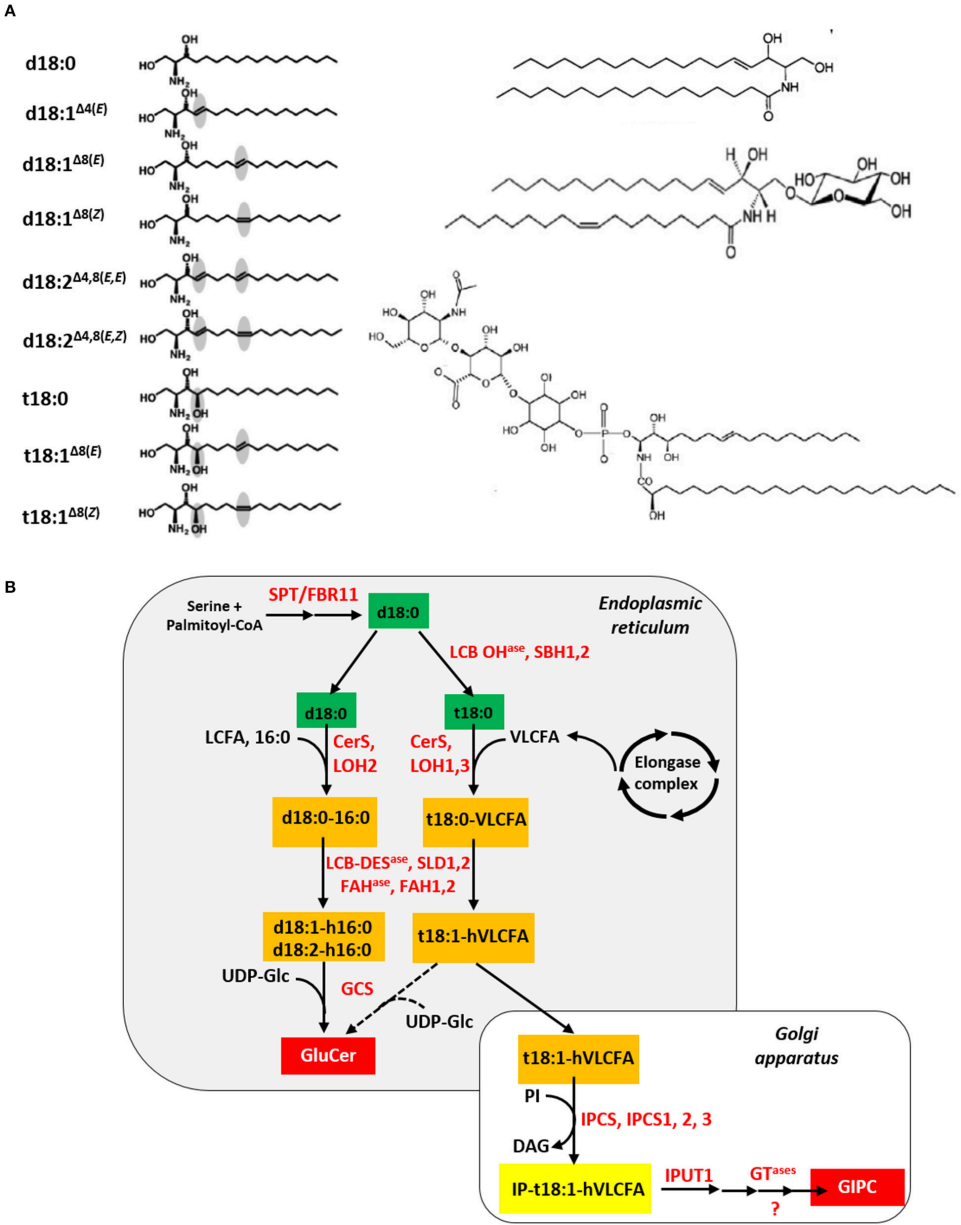
**Plant sphingolipid synthesis**. **(A)** The four classes of plant sphingolipids (from Cacas et al., 2012a). The left panel displays the nine molecular species of long-chain bases found in plants, from top to bottom: sphinganine/dihydrosphingosine, d18:0; sphingosine/sphing-4(*trans*)-enine, d18:1^Δ4(*E*)^; sphing-8(*trans*)-enine, d18:1^Δ8(*E*)^; sphing-8(*cis*)-enine, d18:1^Δ8(*Z*)^; sphinga-4,8(*trans, trans*)-dienine, d18:1^Δ4, 8(*E, E*)^; sphinga-4,8(*trans, cis*)-dienine, d18:1^Δ4, 8(*E, Z*)^; phytosphingosine/4-hydroxysphinganine, t18:0; 4-hydroxysphing-8(*trans*)-enine, t18:1^Δ8(*E*)^; 4-hydroxysphing-8(*cis*)-enine, t18:1^Δ8(*Z*)^. On the right panel (from top to bottom) are showed a ceramide (sphing-4(*trans*)-enine-*N*-octadecanoic acid), a glucosylceramide (Glucosyl-O-β-ceramide (sphing-4(*trans*)-enine-*N*-octadec-9(*cis*)-enoic acid)) and a glycosyl-inositolphosphoryl-ceramide (N-acetylglucosamine-glucuronic acid-inositolphosphoryl-ceramide (4-hydroxysphing-8(*cis*)-enine-*N*-tetracosanoic acid)). **(B)** *In situ* simplified view of the plant sphingolipid biosynthesis pathway. Except for the serine and palmitoyl-CoA precursors, sphingolipid metabolites appear in colored rectangles: green for long-chain bases, orange for ceramides and red for final products like glucosylceramides (GluCer) and glycosyl-inositolphosphoryl-ceramides (GIPC). Nomenclature for ceramide is as follows: for instance, d18:0-16:0 indicates that the long-chain base corresponds to sphinganine and the fatty acid is a palmitoyl moiety, respectively. Enzymes are written in red. Abbreviations: CerS, ceramide synthase; DAG, diacylglycerol; FAH^ase^, fatty acid hydroxylase; GCS, glucosylceramide synthase; GT^ase^, glycosyl-transferase; IPCS, inositolphosphoryl-ceramide synthase; IPUT1, INOSITOLPHOSPHORYL-CERAMIDE GLUCURONOSYL-TRANSFERASE 1; LCB, long-chain base; LCB DES^ase^, LCB desaturase; LCB OH^ase^, LCB hydroxylase; LCFA, long-chain fatty acid; LOH, LAG ONE HOMOLOG; PI, phosphatidylinositol; SPT/FBR11, serine palmitoyl-CoA transferase/FUMONISIN-RESITANT 11; SLD1,2, SPHINGOLIPID LCB Δ8 DESAUTRASE 1,2; UDP-Glc, uridine diphosphate-glucose; VLCFA, very-long-chain fatty acid.

Plant sphingolipids encompass four major classes: long-chain bases (LCB), ceramides (Cer), glucosylceramides (GluCer), and more complex glycosylated sphingolipids, known as glycosyl-inositolphosphoryl-ceramides (GIPC) (Figure 2A). Apart from GIPC, the synthesis of which is initiated in the ER and completed in the Golgi apparatus (GA), the three other classes are produced in the ER (Figure 2B). Neo-synthesis of LCB results from the condensation of serine and palmitoyl-CoA moieties catalyzed by a protein complex, so-called the serine-palmitoyl-CoA transferase (SPT). Subsequent reduction of the SPT product results in the synthesis of sphinganine, the precursor of the eight other LCB found in plants. Ceramide synthases (CerS), encoded by a multigenic family named after the yeast protein LAG ONE HOMOLOG (LOH), are responsible for the formation of the amide bond that links (V)LCFA to LCB, leading to Cer formation. Ceramides can then be used as backbone for the production of GluCer and GIPC by addition of a glucose molecule or an inositolphosphoryl group followed by one or several glycosylation steps, respectively (Markham et al., 2013). Noteworthy, it can be inferred, on the basis of their biosynthetic pathway (Li-Beisson et al., 2010), and additional data (Pata et al., 2010; Cacas et al., 2016c), that sphingolipids contain most VLCFA produced in leaves.

### Scenario 1: interplay between very-long-chain fatty acids and the ceramide and LCB signals

Schematically, GIPC represent two third of total sphingolipids within photosynthetic plant cells whereas Glucer accounts for the other third (Markham et al., 2006). This is coherent with their role as structural membrane elements. By contrast, free Cer and LCB, as intermediate metabolites, are weakly present in leaf organs (Markham et al., 2006). Defining genuine molecular signals as locally- and timely-produced molecules that act at infinitesimal concentrations, it might not be surprising that the signaling function of both LCB and ceramides under stressful conditions could be conserved across kingdoms. Even though exact molecular substratum for sphingolipid control of cell fate is far from being deciphered, it is assumed that, in plants, like in animals, accumulation of free Cer or LCB would kill cells whereas that of their phosphorylated counterparts would have survival-promoting effects in response to stress. In plants, when chemically-perturbed or genetically-disrupted, most steps of the sphingolipid biosynthesis pathway can lead to conditional cell death phenotypes or spontaneous pathogen resistance-mimicking hypersensitive-like foliar lesions. These observations can be tentatively explained by a disequilibrium of the tightly-regulated intracellular balance between unfettered LCB and LCB-phosphate (LCB-P). Compelling evidence for this notion was provided by exogenous LCB application, the use of the mycotoxin fumonisin B1 that inhibits CerS and mutation (*fumonisin-resistant 11, fbr11*) in a subunit of the LCB-forming enzyme SPT (Alden et al., 2011; Berkey et al., 2012). Another crucial regulatory node may also rely on the Cer/Cer-phosphate (Cer-P) ratio, as substantiated by genetic data regarding the ceramide kinase ACCELERATED CELL DEATH 5 (Liang et al., 2003), the Cer-P transferase ACCELERATED CELL DEATH 11 (Simanshu et al., 2014) and inositolphosphoryl-ceramide synthase (IPCS) (Wang et al., 2008).

Recent work addressing *Arabidopsis* CerS specificity toward FA chain length *in vitro* established that LOH1/3 preferentially use VLCFA as substrates whereas LOH2 rather forms sixteen carbon-long fatty acid (16:0)-containing Cer (Luttgeharm et al., 2016). While LOH1/3 overexpressing lines showed only little changes in their sphingolipid profiles, a strong enrichment in Cer molecular species with 16:0 FA was recorded for those that overexpressed LOH2. Additional phenotypical traits of the latter overexpressor plants were reminiscent of lesion-mimic mutants that exhibit enhanced disease resistance and develop hypersensitive cell death symptoms under restrictive environment, despite the absence of pathogens. These traits included increased salicylic acid concentrations and *PATHOGENESIS-RELATED 1* (PR1) gene expression, localized programmed cell death and severe dwarfism (Luttgeharm et al., 2015). Consistently, *loh1* null mutants displayed discrete spontaneous foliar lesions correlated with strong constitutive *PR1* expression and significant elevation in 16:0-containing Cer and GluCer contents (Ternes et al., 2011). Although FA-mediated structural effects cannot totally be ruled out for explaining the aforementioned phenotypes (see below), these data may also pinpoint the importance of the aliphatic chain length present in Cer destined to signaling purposes, suggesting the occurrence of a supplementary regulatory level to that driven by the solely balance of phosphorylated/non-phosphorylated metabolites. In line with this postulate, it is thus tempting to speculate that stress-induced rise in VLCFA concentrations might alter intracellular Cer pool composition and, subsequently, impact related signaling routes. Moving further this way, it is also plausible that, following stress exposure, quantitative and/or qualitative modifications of VLCFA pool indirectly influence LCB synthesis due to metabolic reorientation, as suggested by the analysis of LCB hydroxylase mutants (Chen et al., 2008). The existence for two *Arabidopsis* sphingosine kinases with sharply different substrate specificity also reinforces the idea that both VLCFA and LCB chains could matter when it comes to signaling stress (Guo et al., 2011). Since the *Arabidopsis* MITOGEN-ACTIVATED PROTEIN KINASE 6 (MPK6) has been recently described as a downstream effector of sphingolipid-induced cell death (Saucedo-García et al., 2011), it could represent a privileged target for investigating the interplay between VLCFA and LCB/Cer-contingent signaling under stressful conditions.

### Scenario 2: the membrane trafficking link

The plant endomembrane system (ES) is a complex, dynamic and intricate membrane-composed web that encompasses the ER, Golgi apparatus (GA), trans-Golgi network (TGN), the endocytic, vacuolar, and autophagic compartments, the plasma membrane (PM) and all vesicles that shuttle in between these organelles. It provides infrastructure for the secretory pathway which is dedicated to both protein and lipid sorting (Cacas, 2010). Apart from its crucial role in maintaining cellular homeostasis, the ES has also recently emerged as an essential component of plant tolerance to stress (for review, see Cacas, 2015) and, this may be linked to VLCFA-containing sphingolipids, like GluCer and GIPC.

Early indirect insights into a potential relationships between GluCer and protein trafficking came from clinical studies focused on molecular mechanisms underpinning Gaucher disease. A bench of mutations that totally or partially invalidate the two glucocerebrosidases-encoding genes—involved in lysosomal degradation of GluCer—was found to cause ER to dysfunction, leading to enzyme sorting impairment (Yu et al., 2007). In plants, regulation of GluCer homeostasis is as essential as in animal models. In *Arabidopsis*, only one gene codes for the ER-localized glucosylceramide synthase, or GCS. Null *gcs* mutants fail to develop beyond seedling stage and are defective for organogenesis. In addition, *gcs* (−/−) cells display altered GA morphology indicative of a probable perturbed cell secretion activity (Msanne et al., 2015). Accordingly, ground-breaking work carried out by the team of Dr. Moreau (CNRS, Bordeaux, France) pointed out that chemical blunting of GCS activity resulted in (i) GA disaggregation into vesicles, (ii) reduced externalization of an apoplastic fluorescent protein ectopically expressed (N-SecYFP), and (iii) both mislocalization and secretion diminishment of the PM-located H^+^-ATPase PMA4 (Melser et al., 2010). Transient overexpression of the two latter proteins (N-SecYFP and PMA4) in a WT genetic background was further reported to augment sterol and GluCer contents whereas that of soluble proteins and membrane proteins which do not traffic beyond GA was unable to do so (Melser et al., 2010). Combined, these findings put forward the case for GluCer, along with sterols, as potent protein sorting mediators in the late secretory pathway. Two main explanations can be envisaged in this context. On the one hand, one can assume that GluCer and sterols exerts their function through a chaperone-like activity, stabilizing native structure of specific integral membrane cargo proteins that they escort from GA to PM. On the other hand, it has been proposed that glycosphingolipids could impose positive curvature to membranes, thereby facilitating vesicle fusion (Barth et al., 2010; Molino et al., 2014). In this regard, the few data that are currently available in the literature do not allow discriminating among these two hypotheses yet.

Joined study between our team (Faure's lab, INRA, Versailles, France) and that of Markham (Danforth Plant Science Center, Saint Louis, Missouri, USA) documented the impact of *loh* mutations on *Arabidopsis* root architectural modifications in relation with sphingolipid profile and secretion of PM-resident proteins (Markham et al., 2011). As corroborated latter on by Luttgeharm et al. (2016), it was demonstrated that double *loh1/loh3* mutants overaccumulated 16:0-containing complex glycosphingolipids at the expense of VLCFA-containing ones, reflecting the substrate specificity of the remaining CerS activity borne by LOH2. Remarkably, this marked trend was correlated with PM-targeting default for two auxin carrier proteins; the latter of which being characterized by a loss of cell polarity, a strong inhibition of hormonal transport and the absence of lateral root initiation at macroscopic level. Again, experimental data argued in favor of a post-Golgi trafficking defect when sphingolipid synthesis was manipulated (Markham et al., 2011). Together with those of Melser's work, our results provide unequivocal evidence for the requirement of VLCFA-containing sphingolipids for protein transport, even though the respective contribution of GluCer and GIPC in this process could not have been ascertained. Back to signaling topic, it is possible that VLCFA anabolism adjustment participate in accommodating cell secretory activity to challenging environmental conditions. In fact, several published examples already indicate that effective intracellular membrane trafficking is necessary for transducing specific protein-based signals during plant immunity (for reviews, see Berkey et al., 2012; Teh and Hofius, 2014). Among them can be cited the immune receptor EIX2 (ETHYLENE-INDUCING XYLANASE 2) from tomato plants, the endosomal internalization of which is necessary for mounting proper defense response (Sharfman et al., 2011). Another striking example is that of the relocalization of the RPW8 resistance protein to extrahaustorial membranes at the host-pathogen interface in response to specific fungi and oomycetes (Wang et al., 2009). Moreover, numerous protein regulators that control cell death outcome in response to abiotic and biotic stress are distributed along the ES (Cacas, 2015). This sustains the idea that VLCFA could define a late secretory pathway dedicated to some stress signaling components. One of the main challenges in next future will be to understand the role of one such path under abiotic constraints. Distinguishing how VLCFA could, respectively, influence antimicrobial protein burden to be excreted and transport of specific regulatory proteins following pathogen infection will probably represent a difficult task too.

### Scenario 3: the membrane microdomain hypothesis

Membrane microdomains can be defined as islands composed of lipids and proteins that laterally segregate from the rest of the PM. They are highly enriched in sterols, sphingolipids and signaling proteins (Boutté and Grebe, 2009). Their size was described to fit nanometer to micrometer scales. Originally named raft, microdomains were identified in mammalian systems where they notably act as platforms responsible for the launching of apoptotic and inflammation signaling cascades (Malorni et al., 2007; George and Wu, 2012). First experimental evidence for their occurrence in plants was provided by biochemical approaches based on the purification of detergent-insoluble membranes (DIM) through floatation on step sucrose gradient (Mongrand et al., 2004). Since then, accumulation of pharmacological, proteomic, microscopy, and genetic data ended the controversy on plant microdomain existence. Thus, it is now broadly accepted that DIM do not represent functional equivalents of microdomains, but rather constitutes one way of assessing their chemical composition (Cacas et al., 2012a).

Previously, a strong enrichment in tri-hydroxylated LCB in PM fractions purified from two plant species was reported (Borner et al., 2005; Lefebvre et al., 2007). Given that this class of LCB is mainly encountered in GIPC (for review, see Pata et al., 2010), this led the plant lipid community to the reasonable hypothesis that GIPC reside, for the most part, in PM. Having established methods for purifying GIPC and characterizing their composition (Buré et al., 2011; Cacas et al., 2012b), we tested for this assumption using tobacco plants and cell cultures. Not astonishingly, we found that (i) tobacco GIPC contain the large majority of the intracellular VLCFA pool, and (ii) VLCFA moieties engaged in these lipids were predominantly hydroxylated on carbon position 2 (noted hVLCFA). Exploiting this unique opportunity for probing GIPC repartition within the ES uncovered a marked hVLCFA gradient along the secretory pathway that reaches an optimum in DIM fractions. Further investigations brought to light that GIPC amount to approximately 60 mole % of total DIM lipids; the polyglycosylated forms being only present in the external hemi-layer and clustering in 35 nm-sized microdomains. Combined biophysical and modeling strategies also showed that hVLCFA could strongly interact with sterols and interdigitate between the two membrane leaflets, which likely explains the synergistic effect of GIPC and sterol in structuring membrane *in vitro* (Cacas et al., 2016c). Hence, in addition to their postulated role in protein sorting at the TGN, glycosphingolipids may also be involved in stress response with respect to their lateral segregation within PM.

Nagano et al. (2009, 2012) shed light on possible links between hVLCFA and stress acclimation. Working on the conserved family of ER-resident cell death regulators, known as BAX INHIBITORS (BI), the authors showed that At-BI1 interacts with the electron donor, cytochrome b5, the latter of which can in turn associate with FATTY ACID HYDROXYLASE1 (FAH1) catalyzing the hydroxylation of VLCFA. Overexpression of At-BI1 was also correlated with higher hVLCFA contents and decreased cell death under stressful conditions. Conversely, knock-down *FAH1* plants displayed decreased hVLCFA amounts and enhanced sensitivity to hydrogen peroxide, suggesting that At-BI1 protects cells by activating FAH1. Now, given that hCer exhibit pro-survival properties in animal cells (Young et al., 2013), one can hypothesize that the hCer/Cer balance under the control of BI1/FAH1 dictates the progression rate of hypersensitive foliar lesions in response to pathogen attack. This attractive theory cannot, however, justify by itself MYB30-driven transcriptional induction of the elongase complex genes (Raffaele et al., 2008) and the resulting massive increase in VLCFA concentrations reported in this context. Alternatively, one can envisage that modulating hVLCFA synthesis could affect GIPC composition and/or concentration and, consequently, impact raft signaling events. *In vivo* and *in vitro* experiments have proven that lateral segregation of membrane proteins is dependent on that of lipids, and vice versa (for review, see Volmer and Ron, 2015). In animal systems, recruitment or disassembly of signaling actors can be achieved through respective coalescence or dissociation of microdomains, thereby provoking initiation or termination of signaling cascades at the PM (Malorni et al., 2007; George and Wu, 2012). A comparable situation has already been proposed to take place in *BI1*-overexpressing transgenics (Ishikawa et al., 2015) and during plant innate immunity (Keinath et al., 2010). In addition, both biotic and abiotic stresses are known to provoke changes in protein content of microdomains (Minami et al., 2009; Stanislas et al., 2009).

Findings that elongase complex-encoding genes are under transcriptional control upon environmental cues (Joubès et al., 2008; Raffaele et al., 2008; Xie et al., 2015) implies that modulation of hVLCFA steady-state levels is implicated in a secondary signaling wave, possibly regulating microdomain-coordinated events. Indeed, for transcriptional reprogramming to occur, stress perception must be completed and signal transduction engaged. This is quite distinct from, but not incompatible with the regular picture documented in the mammalian literature where external constraints are generally described to promote rapid relocalization of sphingolipid-modifying enzymes to microdomains, freeing Cer or LCB moieties and, *per se*, generating primary signals relayed by downstream effectors. Actually, one can expect that both situations could cohabit in the same challenged plant cell with different timing. In this case, raft sphingolipids could feature a reservoir of signals to be mobilized early following stress application, as sustained by the recent discovery of sphingolipase D activities in plants (Tanaka et al., 2013). Once initiated, such signaling cascades would contribute to activate hVLCFA neo-synthesis, ultimately fine-tuning microdomain composition. The latter phenomena could either benefit to intercellular communication or simply operate as a negative feedback that abrogate the production of signaling molecules by microdomains. Testing for this seductive concept will require a careful *in situ* dosage of hVLCFA-containing sphingolipids over time. With the recent advances in mass spectrometry-based chemical imaging, this deadlock should be broken in a close future.

## Tentative model–puzzling out very-long-chain fatty acid-contingent signaling pathways

**Figure 3:**
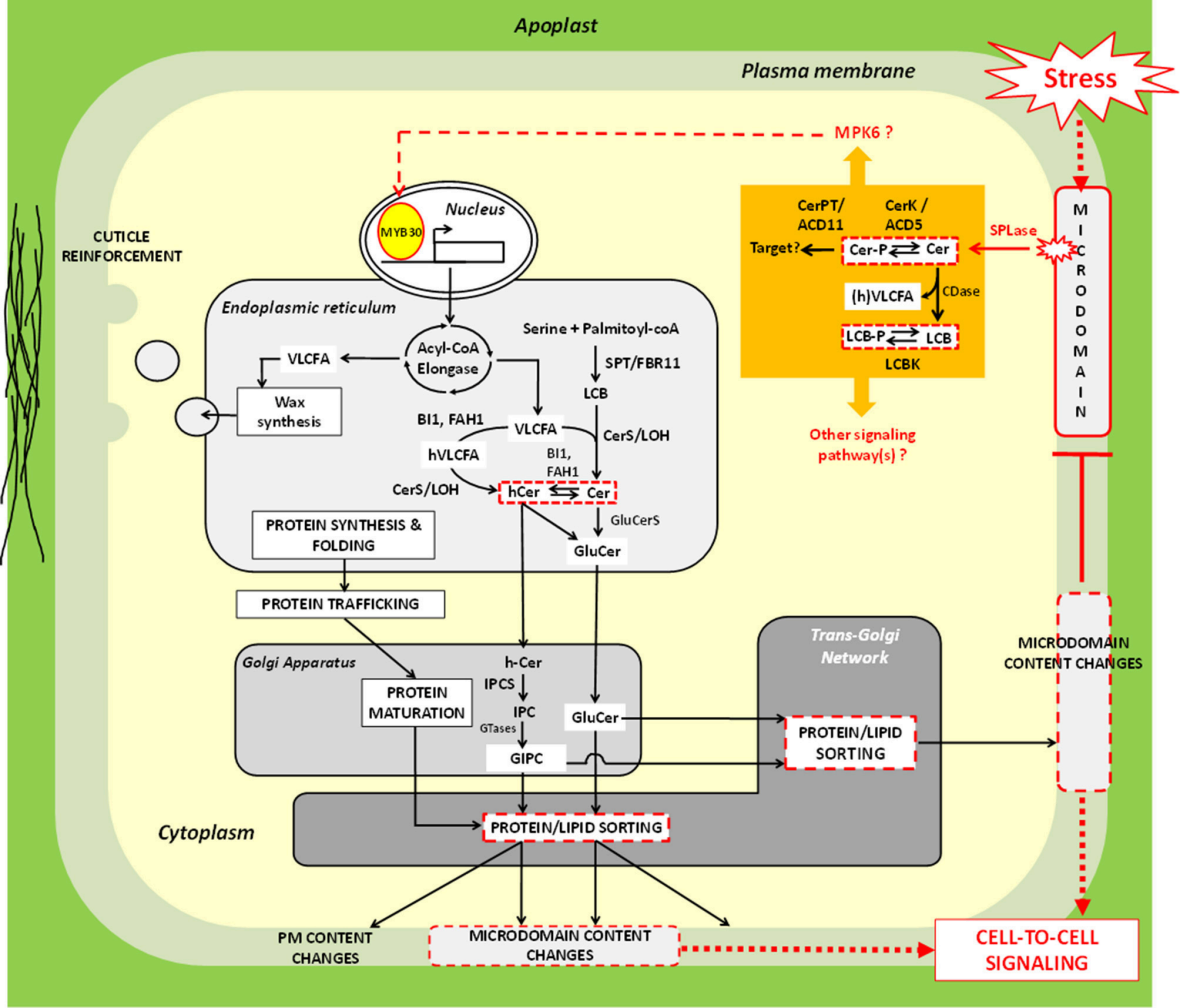
**Molecular model explaining how very-long-chain fatty acids could participate in stress signaling response in plant cells**. In mammalian systems, extrinsic cues can be perceived at the plasma membrane by means of microdomains. A comparable hypothesis can be emitted for plant models. It is possible that “sphingolipase D” (SPLase) like the one identified by Tanaka et al. (2013) is recruited to microdomains following stress exposure, and releases ceramide (Cer) molecules from complex glycosphingolipids *in situ*. Free Cer could either directly act as signals or be processed into Cer-P by the kinase ACCELERATED CELL DEATH 5 (ACD5). The ceramide-1-phosphate tranferase ACCELERATED CELL DEATH 11 (ACD11) may participate to this signaling cascade as well, though its exact mode of action remains to be clarified. In addition, Cer could also be hydrolyzed by ceramidase (CDase) into LCB that can, in turn, be phosphorylated; one such activity have indeed been documented in plants (Pata et al., 2010). Although little is still known about molecular actors that relay LCB/Cer signals (orange part of the figure), the work of Saucedo-García et al. (2011) identified MITOGEN-ACTIVATED PROTEIN KINASE 6 (MPK6) as a good candidate for exerting this function. The transcription factor MYB30 represents a potential downstream target of sphingolipid-induced phosphorylation events, since it was found to up-regulate acyl-CoA elongase genes in response to environmental cues. Resulting very-long-chain fatty acid (VLCFA) production could then be utilized for strengthening cuticle, especially epicuticular wax. Alternatively, VLCFA could be incorporated into sphingolipids. Modifications of sphingolipid composition and/or level can impact protein sorting at the TGN and, therefore, probably modulate targeting of specific stress responsive signaling proteins to PM. From this postulate, it seems coherent to envisage that modifications of the secreted lipids and proteins influence PM lateral segregation. Expected consequences of this segregation phenomenon could be changes in microdomain content that could feature extracellular signaling process(es) and/or negative feedback regulation. Of note, elements in the picture that represent regulatory nodes involving VLCFA (Hypotheses 1-3) are delineated by red continue or discontinued lines. Red discontinued arrows indicate steps which has not been experimentally demonstrated. For additional abbreviations, refer to the legend of Figure 2.

Although the involvement of VLCFA in stress response is not contestable, interpretation of this experimental fact may remain delicate in light of the currently available data. Pleiotropic consequences of VLCFA level alterations also render this task complicated. Based on the observation that VLCFA are highly enriched in sphingolipids, we have, however, suggested, and explored three non-exclusive, molecular scenarii to tentatively explain how insoluble molecules —like VLCFA—could participate in stress signaling response. These hypotheses, which are experimentally testable, are summarized in a model presented in the Figure 3. By analogy with animals systems, it is envisaged that stress perception could trigger recruitment of yet-to-be cloned “sphingolipases” to microdomains. Enzymatically-released Cer(-P) skeletons could then either directly serve as signals or be further processed, activating a potent downstream effector, the kinase MPK6. One possible target of this phosphorylation cascade could be the transcription factor MYB30, which is known to up-regulate expression of the elongase complex-encoded genes upon pathogen attack and hypoxia. As also supported by several studies, changes in the composition and/or level of VLCFA-containing lipids impact protein sorting at the TGN. This could represent a potential regulatory mechanism whereby targeting of specific signaling proteins to PM could be spatio-temporally modulated depending on stressed cell requirements. Adding a supplemental layer of regulation, these changes in sphingolipids certainly alter microdomain content, and consequently, should also influence PM-coordinated signaling events. Beyond the response plasticity conferred to plant cells by a dual lipid/protein-based rheostat, this model raises the interesting question as to how this molecular scheme contributes to stress acclimation. Is this linked to intercellular communication, negative feedback control of microdomain-dependent signaling or both?

## Author contributions

AD provided Table 2, JC defined hypotheses, wrote the manuscript, and drew figures.

### Conflict of interest statement

The authors declare that the research was conducted in the absence of any commercial or financial relationships that could be construed as a potential conflict of interest.

## Abbreviations

ACBP: Acyl-CoA Binding Protein
ACD5/11: ACCELERATED CELL DEATH 5/11
BI1: BAX INHIBITOR 1
Cer: ceramide
Cer-P: ceramide phosphate
CerS: ceramide synthase
CoA: coenzyme A
DIM: detergent-insoluble membranes
ECR: enoyl-CoA reductase
EIX2: ETHYLENE-INDUCING XYLANASE 2
ER: endoplasmic reticulum
ES: endomembrane system
FAH1: fatty acid hydroxylase 1
FFAR/GPAR: FREE FATTY ACID RECEPTORS/G PROTEIN-COUPLED RECEPTORS
FA: fatty acid
FAH: fatty acid hydroxylase
FAS: fatty acid synthase
GA: Golgi apparatus
GFP: green fluorescent protein
GIPC: glycosyl-inositolphosphoryl-ceramide
GluCer: glucosylceramide
HCD: hydroxyacyl-CoA dehydratase
(h)VLCFA: (2-hydoxy-)very-long-chain fatty acid
IPCS: inositolphosphoryl-ceramide synthase
KCR: β-ketoacyl-CoA reductase
KCS: β-ketoacyl-CoA synthase
LCB: long-chain base
LCB-P: long-chain base phosphate
LOH: LAG ONE HOMOLOG
MPK6: MITOGEN-ACTIVATED PROTEIN KINASE 6
NADPH: Nicotinamide Adénine Dinucléotide Phosphate
PM: plasma membrane
PR1: *PATHOGENESIS-RELATED 1*
SPT: serine-palmitoyl-CoA transferase
TGN: trans-Golgi network.
